# Bayesian penalised likelihood reconstruction (Q.Clear) of ^18^F-fluciclovine PET for imaging of recurrent prostate cancer: semi-quantitative and clinical evaluation

**DOI:** 10.1259/bjr.20170727

**Published:** 2018-01-19

**Authors:** Eugene J Teoh, Daniel R McGowan, David M Schuster, Maria T Tsakok, Fergus V Gleeson, Kevin M Bradley

**Affiliations:** 1 Department of Radiology, Churchill Hospital, Oxford University Hospitals NHS Trust, Oxford, UK; 2 Department of Oncology, University of Oxford, Oxford, UK; 3 Department of Radiation Physics and Protection, Churchill Hospital, Oxford University Hospitals NHS Trust, Oxford, UK; 4 Department of Radiology and Imaging Sciences, Emory University, Atlanta, GA, USA

## Abstract

**Objective:**

^18^F-Fluciclovine (FACBC) is an amino acid PET radiotracer approved for recurrent prostate cancer imaging. We investigate the use of Bayesian penalised likelihood (BPL) reconstruction for ^18^F-fluciclovine PET.

**Methods:**

15 ^18^F-fluciclovine scans were reconstructed using ordered subset expectation maximisation (OSEM), OSEM + point spread function (PSF) modelling and BPL using β*-*values 100–600. Lesion maximum standardised uptake value (SUV_max_), organ SUV_mean_ and standard deviation were measured.

Deidentified reconstructions (OSEM, PSF, BPL using β200–600) from 10 cases were visually analysed by two readers who indicated their most and least preferred reconstructions, and scored overall image quality, noise level, background marrow image quality and lesion conspicuity.

**Results:**

Comparing BPL to OSEM, there were significant increments in lesion SUV_max_ and signal-to-background up to β400, with highest gain in β100 reconstructions (mean ΔSUV_max_ 3.9, *p* < 0.0001). Organ noise levels increased on PSF, β100 and β200 reconstructions. Across BPL reconstructions, there was incremental reduction in organ noise with increasing β, statistically significant beyond β300–500 (organ-dependent). Comparing with OSEM and PSF, lesion signal-to-noise was significantly increased in BPL reconstructions where β ≥ 300 and  ≥ 200 respectively.

On visual analysis, β 300 had the first and second highest scores for image quality, β500 and β600 equal highest scores for marrow image quality and least noise, PSF and β 200 had first and second highest scores for lesion conspicuity. For overall preference, one reader preferred β 300 in 9/10 cases and the other preferred β 200 in all cases.

**Conclusion:**

BPL reconstruction of ^18^F-fluciclovine PET images improves signal-to-noise ratio, affirmed by overall reader preferences. On balance, β300 is suggested for ^18^F-fluciclovine whole body PET image reconstruction using BPL.

**Advances in knowledge:**

The optimum β is different to that previously published for ^18^F-fluorodeoxyglucose, and has practical implications for a relatively new tracer in an environment with modern reconstruction technologies.

## Introduction

The optimisation of Bayesian penalised likelihood (BPL) reconstruction (Q.Clear, GE Healthcare) for ^18^F-fluorodeoxyglucose (FDG) whole body PET,^1^ and its effect on evaluating various clinical entities on ^18^F-FDG PET/CT have been reported.^2–5^ BPL runs to effective convergence and includes point spread function (PSF) modelling while controlling noise through the use of a penalty term (β), which achieves greater noise reduction as β is increased.^1^ It has been shown to improve lesion signal-to-noise ratio (SNR) on ^18^F-FDG PET compared to widely utilised ordered subset expectation maximisation (OSEM) reconstruction, particularly in small, subcentimetre abnormalities.^2^ Our institution currently uses BPL reconstructed images (β = 400)^1^ for the clinical interpretation of all ^18^F-FDG whole body PET/CT studies.

The burgeoning repertoire of non-FDG PET tracers coupled with progressive adaptation of modern image reconstruction technology demands tracer-specific optimisation of its use. This is due to inherent variation of physiological distribution and degree of pathological uptake observed between different tracers. More importantly, the practice of image optimisation remains paramount to maintaining duty of care to the patient.

^18^F-Fluciclovine (anti-1-amino-3-fluorocyclobutane-1-carboxylic acid/FACBC) is a synthetic amino acid PET radiotracer with expectant clinical demand, followed recent approvals by the Food and Drug Administration and European Medicines Agency as a diagnostic agent for detection of recurrent prostate cancer.^6, 7^ The aim of this study was to compare BPL to standard PET reconstruction of ^18^F-fluciclovine images and determine the optimum penalisation factor (β) for clinical use of BPL in ^18^F-fluciclovine imaging.

## Patients and methods

### Case selection

This study was approved by the South Central Berkshire Research Ethics Committee. Fifteen ^18^F-fluciclovine whole body scans performed consecutively between October 2015 and August 2016 for biochemical recurrence of prostate cancer were retrospectively selected. The median patient weight was 77.3 kg (range 56–102 kg).

### ^18^F-Fluciclovine PET/CT imaging protocol

Image acquisition was performed on a 3D-mode time-of-flight GE Discovery 710 PET/CT system (GE Healthcare, Milwaukee, WI). Patients were required to fast for at least 4 h before injection. Imaging commenced 3–5 min post-injection of ^18^F-fluciclovine (327 to 418 MBq) covering the skull base to proximal thighs. PET images were acquired under normal tidal respiration, commencing caudally, for 5 min per bed position for the first two bed positions (over the pelvis), and 3 min per bed position for the remaining acquisition.

### Semi-quantitative analysis

Sinograms of the 15 scans were reconstructed using three different algorithms, each of which used the CT scan for attenuation correction and the same normalisation correction factors with scatter and randoms corrected as has been previously described.^8^ The first algorithm was OSEM (ToF OSEM, VPFX, GE Healthcare), used with 2 i, 24 ss and 6.4 mm filter. The second was using OSEM + PSF, henceforth referred to as PSF (ToF OSEM PSF, 3 i, 24 ss, 2 mm filter),^1^ and the third was BPL using a range of penalisation factors (β): 100, 200, 300, 400, 500 and 600.

The following parameters were measured on each of the reconstructions: lesion maximum standardised uptake value (SUV_max_), SUV_mean_ and standard deviation (SUV_dev_, noise) of reference organs (marrow, spleen, blood pool, liver). Lesions were defined as small foci of presumed local or distant recurrence. SUV_max_ of each lesion was recorded using a standard volume of interest (VOI) tool. Organ SUVs were measured as follows: marrow–L3 vertebra using a 1.5 cm edge cube VOI, spleen–interpolar region using a 2.0 cm diameter spherical VOI, blood pool–mid-thoracic aorta using a 1.0 cm diameter, 2.0 cm long cylindrical VOI, liver–right lobe of liver using a 3.0 cm diameter spherical VOI. SNR and signal-to-background ratio (SBR) were calculated. Lesion SNR was defined as lesion SUV_max_ divided by marrow SUV_dev_. Lesion SBR was defined as lesion SUV_max_ divided by marrow SUV_mean_. Marrow uptake was used for normalisation based on the moderate and heterogeneous ^18^F-fluciclovine uptake seen in this organ. Organ SNR was calculated as organ SUV_mean_ divided by organ SUV_dev_.

### Statistical analysis

Statistical comparisons were made with reference to both OSEM and PSF, using repeated measures ANOVA with Dunnett’s multiple comparison *post-hoc* testing. Statistical analyses were performed using GraphPad Prism v. 7.0 a (GraphPad Software, La Jolla, CA). Differences in SUV were denoted by “ΔSUV”. *p*-values < 0.05 were considered to be statistically significant. Inter-reader agreement in the visual analysis was evaluated using weighted Cohen’s κ with linear weights. *κ* values were interpreted using guidelines laid out by Landis and Koch.^9^

### Clinical evaluation—reconstruction and visual analyses

Ten of the 15 scans were used for this component of the study. Visual analyses of the OSEM, PSF, BPL β200–600 reconstructions, were performed by two consultants (designated Reader 1 and 2) with dual accreditation in clinical radiology and nuclear medicine, both with more than 10 years of nuclear medicine subspecialty experience. Having previously described similarities between BPL β100 and PSF in (greater) image noise,^1^ reaffirmed by subsequent results of the semi-quantitative analysis, β100 was omitted from the visual analyses.

The reconstructions were labelled A to G in a randomised order, with the CT component available for image fusion and non-attenuation corrected images for reference. Cases were reviewed sequentially, and reconstructions were scored (from 1 to 5) according to four image quality parameters: overall image quality, lesion conspicuity, overall noise level and marrow image quality. Readers were provided with guidance on features which should constitute each score (Table 1). For every reconstruction, a final score for each parameter was derived from the sum of scores given to the relevant parameter for the particular reconstruction. For example, the final score for lesion conspicuity in OSEM would be a sum of the individual scores for this parameter in OSEM reconstructions across the 10 cases.

**Table 1.** Scoring system applied to the four image quality parameters in the visual analysis

**Score**	**Overall image quality/lesion conspicuity**	**Overall noise level (excluding marrow)/background marrow image quality**
1	Not reportable	Non-diagnostic
2	Poor	Numerous heterogeneities throughout entire study, reduced diagnostic quality
3	Satisfactory	Numerous small heterogeneities
4	Good	Minimal heterogeneities
5	Excellent	No significant noise

Readers indicated their most and least preferred reconstruction for each case. Proportions of the highest and lowest ranked reconstructions were calculated for each parameter.

## Results

**Figure 1.**
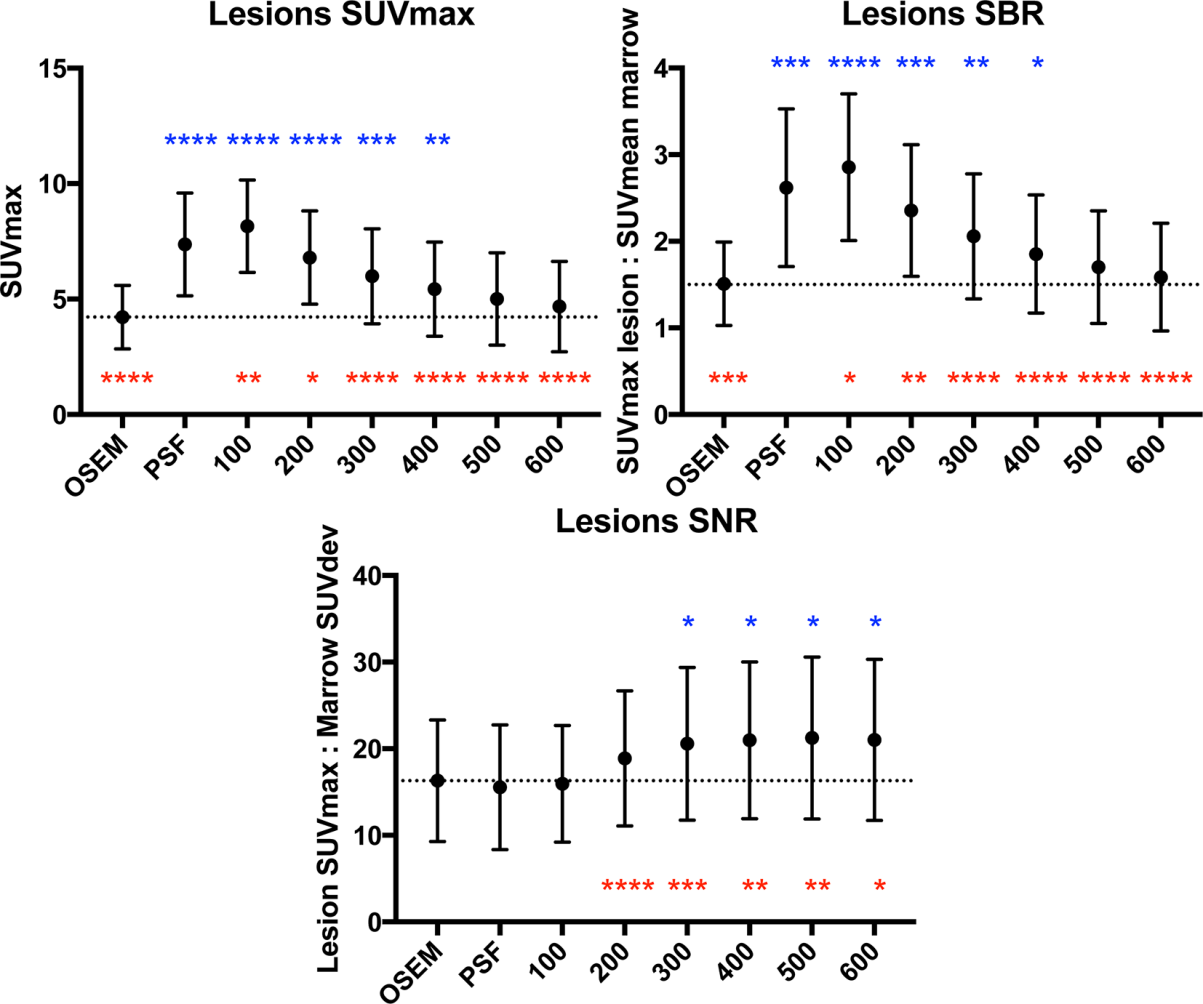
Graphs of lesion SUV_max_, SBR and SNR across the different reconstructions (mean ± standard deviation, dotted line represents mean value on OSEM reconstruction). Statistical comparisons to OSEM are represented above the data points, and comparisons to PSF are represented below the data points (*p < 0.05, **p ≤ 0.01, ***p ≤ 0.001, ****p ≤ 0.0001). OSEM, ordered subset expectation maximisation; SBR, signal-to-background ratio; SNR, signal-to-noise ratio; SUV, standardised uptake value.

**Figure 2.**
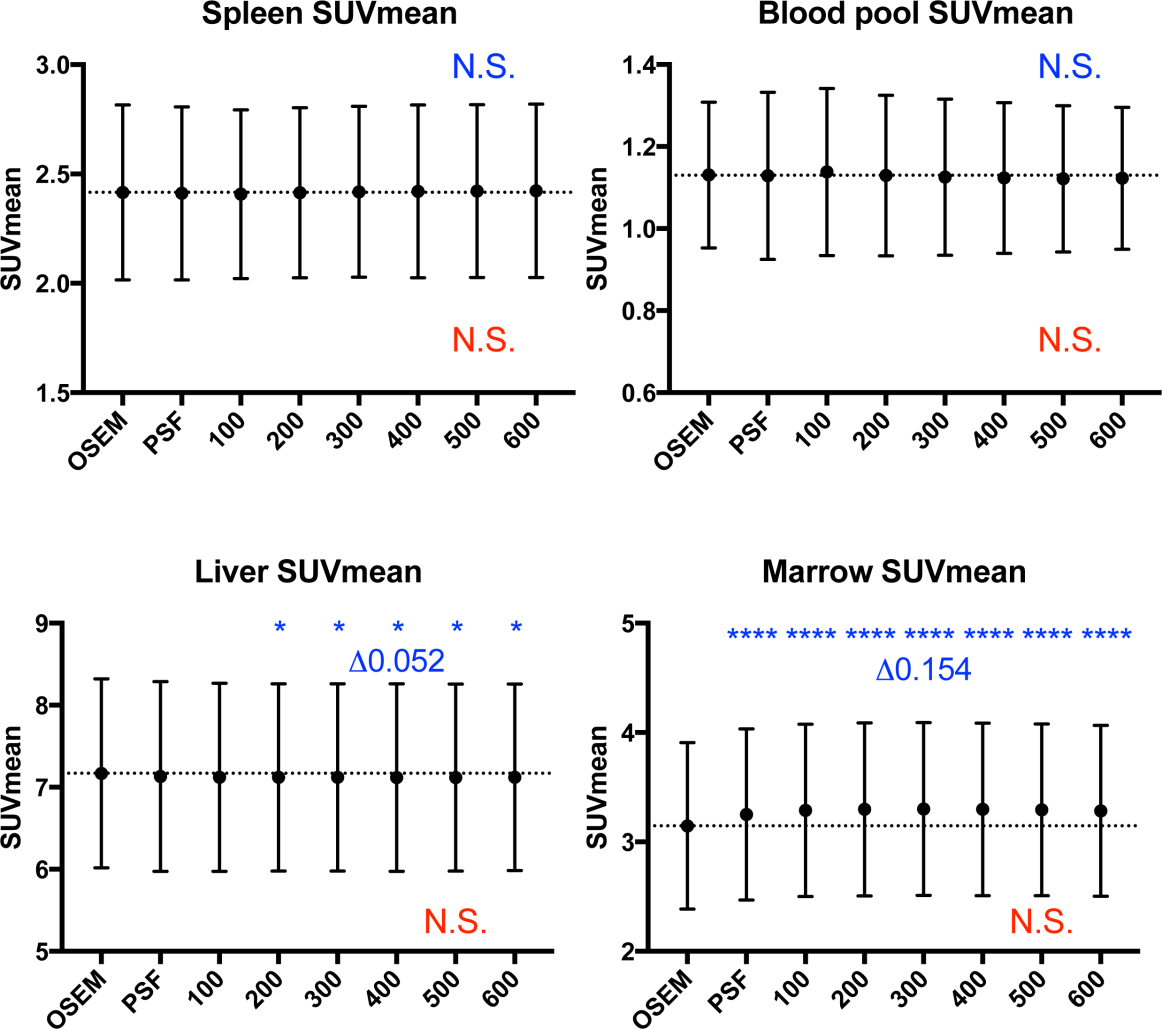
Graphs of organ SUV_mean_ (mean ± standard deviation, dotted line represents mean value on OSEM reconstruction). While there were statistically significant differences between OSEM and the other reconstructions in the liver and marrow (highest ΔSUV_mean_ 0.052 and 0.154 respectively), these were deemed clinically insignificant. Statistical comparisons to OSEM are represented in blue above the data points, and comparisons to PSF are represented in red below the data points (*p < 0.05, ****p ≤ 0.0001). OSEM, ordered subset expectation maximisation; PSF, point spread function; SUV, standardised uptake value.

**Figure 3.**
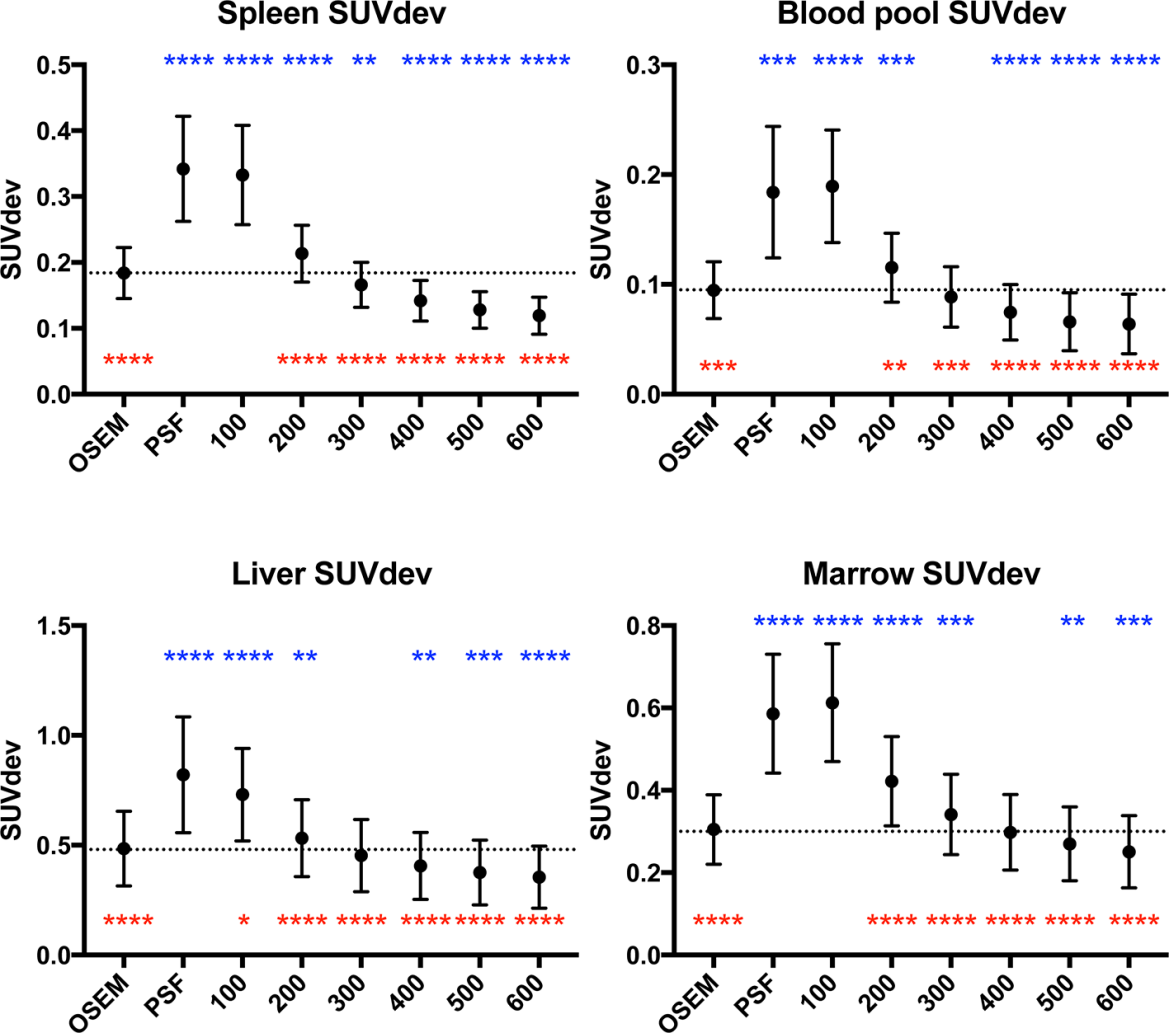
Graphs of organ SUV_dev_ (mean ± standard deviation, dotted line represents mean value on OSEM reconstruction). Statistical comparisons to OSEM are represented in blue above the data points, and comparisons to PSF are represented in red below the data points (*p < 0.05 **p ≤ 0.01, ***p ≤ 0.001, ****p ≤ 0.0001). OSEM, ordered subset expectation maximisation; PSF, point spread function; ; SUV, standardised uptake value.

**Figure 4.**
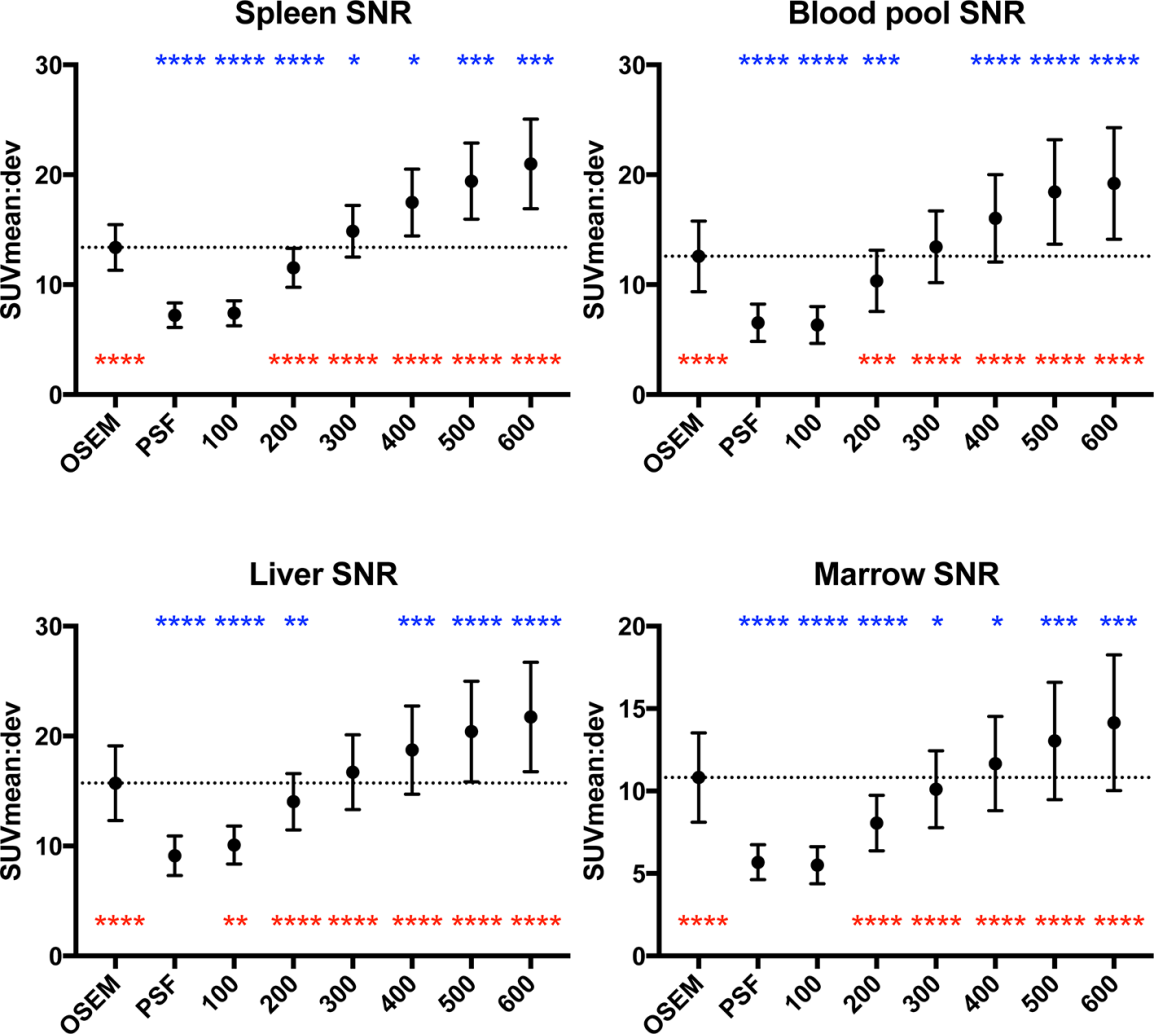
Graphs of organ SNR across the different reconstructions (mean ± standard deviation, dotted line represents mean value on OSEM reconstruction). Statistical comparisons to OSEM are represented in blue above the data points, and comparisons to PSF are represented in red below the data points (*p < 0.05, **p ≤ 0.01, ***p ≤ 0.001, ****p ≤ 0.0001). OSEM, ordered subset expectation maximisation; PSF, point spread function; SNR, signal-to-noise ratio; SUV, standardised uptake value.

**Figure 5.**
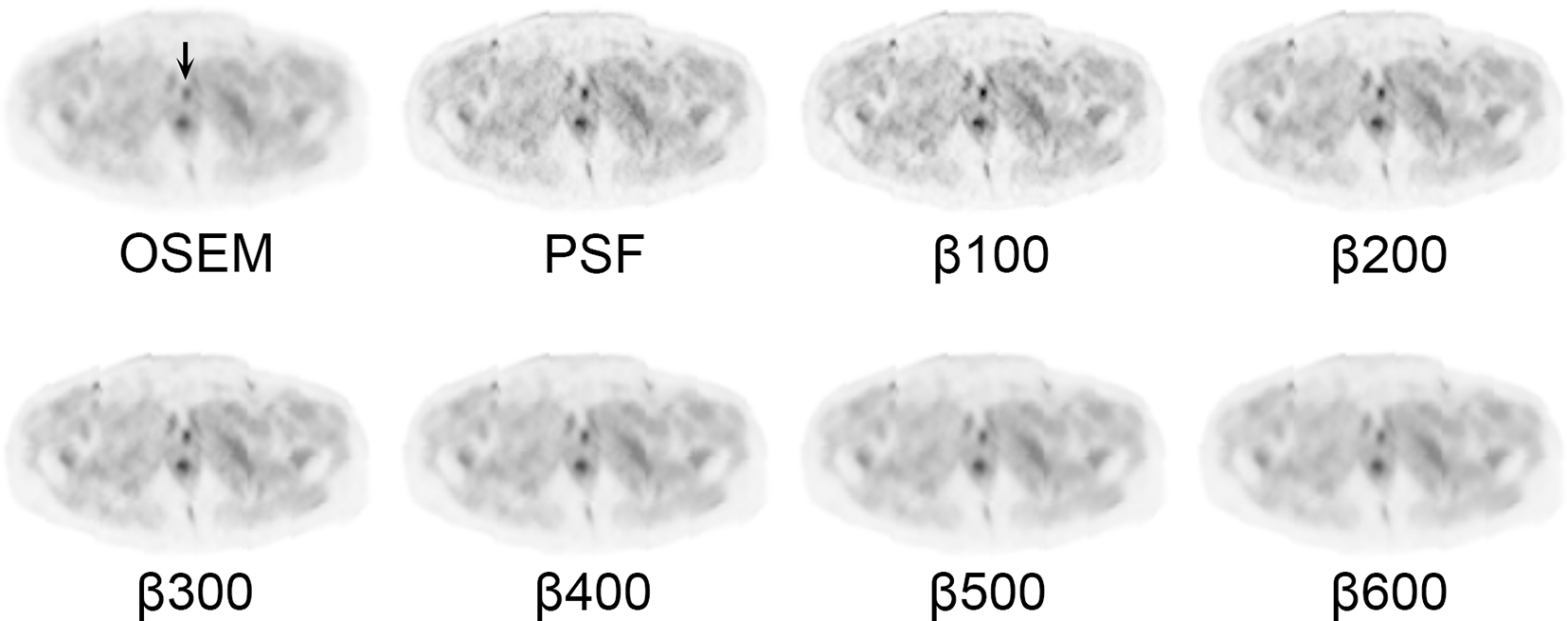
Axial images at the level of the pubic symphysis. Different reconstructions demonstrating a subcentimetre ^18^F-fluciclovine-avid focus of disease (arrow) in the anterior prostatectomy bed. Consistent with the semi-quantitative results, the lesion is rendered more conspicuous on BPL reconstructions with relatively less background noise. All images are displayed on SUV scale 0–6. OSEM, ordered subset expectation maximisation; PSF, point spread function; SUV, standardised uptake value.

Results for the semi-quantitative analyses are summarised in Figures 1–4, and a representative image for each reconstruction is presented in Figure 5. Compared to OSEM, lesion SUV_max_ and SBR increased in β100–400 and PSF, with highest gain in β100 reconstructions (mean ΔSUV_max_ 3.9, *p* < 0.0001) (Figure 1). Lesion SNR was increased in BPL reconstructions compared to OSEM where β ≥ 300 (Figure 1), with no significant intergroup difference between β300–600 (*p* = 0.562). When compared to PSF, lesion SNR was increased in all BPL reconstructions (*p* = 0.0001–0.021), with the exception of β100.

Comparing to OSEM, there was no change in SUV_mean_ of spleen and blood pool across all reconstructions (Figure 2). While such changes in the liver and marrow were statistically significant (Figure 2), these were deemed clinically insignificant (highest ΔSUV_mean_: 0.052 in liver, 0.154 in marrow). There was no change in SUV_mean_ of all the organs when comparison was made to PSF.

Across the BPL reconstructions, there was an incremental reduction in organ noise with increasing β, which compared to OSEM, were significant beyond β300–500 (organ-dependent) (Figure 3). Compared to OSEM, organ noise levels increased on PSF, β100 and β200 reconstructions (Figure 3). This trend was also demonstrated in organ SNR, with statistically significant gains (over OSEM) in marrow, liver and blood pool when β ≥ 400, and the spleen when β ≥ 300 (Figure 4). The opposite trend was observed when comparisons were made to PSF where noise was significantly lower, and SNR significantly higher, in all organs where β ≥ 200 (Figures 3 and 4).

In the clinical evaluation, there was moderate agreement in scores between the two readers [*κ* = 0.48, 95% CI (0.40–0.56)]. β300 had the first and second highest scores (between both readers) for overall image quality, PSF and β200 had first and second highest scores for lesion conspicuity, β500 and β600 had joint highest scores for marrow image quality and overall noise level (*i.e.* least noise) (Table 2). These findings complemented those of the semi-quantitative analyses.

**Table 2.** Scores of image quality parameters in the visual analysis

Reconstruction	Overall image quality		Lesion conspicuity		Overall noise level		Background marrow image quality	
Reader 1	Reader 2	Reader 1	Reader 2	Reader 1	Reader 2	Reader 1	Reader 2
**OSEM**	34	37	19	20	43	36	44	39
**PSF**	27	39	30	29	22	21	21	20
**β 200**	36	50	30	28	33	33	31	29
**β 300**	50	47	27	22	44	39	44	33
**β 400**	45	40	22	22	49	45	49	41
**β 500**	39	30	21	16	50	50	50	50
**β 600**	36	30	19	15	50	50	50	50

OSEM, ordered subset expectation maximisation; PSF, point spread function.

For every reconstruction, a final score for each image quality parameter was derived from the sum of scores given to the relevant parameter for the particular reconstruction.

In terms of overall preferences, Reader 1 preferred β300 in 90% of cases and Reader 2 preferred β200 in all cases. There was a greater polarisation of opinion with regards to least preferred reconstructions, with Reader 1 indicating PSF in 80% and Reader 2 chose β600 in 90% of cases.

## Discussion

The aim of this study was to compare BPL to standard PET reconstruction of ^18^F-fluciclovine images and determine the optimum penalisation factor (β) for clinical use of BPL in ^18^F-fluciclovine whole body imaging. This was addressed using a combination of semi-quantitative and visual analyses performed on a range of reconstructions of clinical scans.

Consistent with prior observations,^1–5^ the majority of BPL reconstructions demonstrated an increase in lesion signal and reduction in noise over OSEM. While lesion signal was higher in the PSF reconstruction compared to BPL reconstructions β ≥ 200, this came at a significant detriment to image noise, which was consistently lower in these BPL reconstructions (β ≥ 200) and widely used OSEM reconstruction (Figure 3). This reinforces observations from our prior work and others,^1, 10^ and is the basis for non-adoption of PSF in our institution when initially released.

The increase in lesion signal (SUV_max_, SBR) compared to OSEM reached statistical significance in the BPL reconstructions up to β400 (Figure 1), while that of noise level showed a decrease in three out of four organs (one statistically significant) at β300 (Figure 3). At β400 and higher, noise was decreased in all organs compared to OSEM (three statistically significant at β400, Figure 3). Accordingly, an increase in lesion SNR was demonstrated in all reconstructions at β300 and higher, with no statistical advantage on semi-quantitative parameters, of one reconstruction over the other beyond this point (Figure 1). These collective findings tentatively placed either β300 or β400 as the optimal BPL reconstruction setting.

The visual analysis proved useful in stratifying this outcome. Between the readers, β300 had the first and second highest scores for overall image quality of clinical scans, while β400 scored second and third highest respectively. This trend was also mirrored in scoring of lesion conspicuity where, after PSF and β200 (which demonstrate a detrimental increase in noise despite signal gain), β300 was scored higher than β400 by both readers.

**Figure 6.**
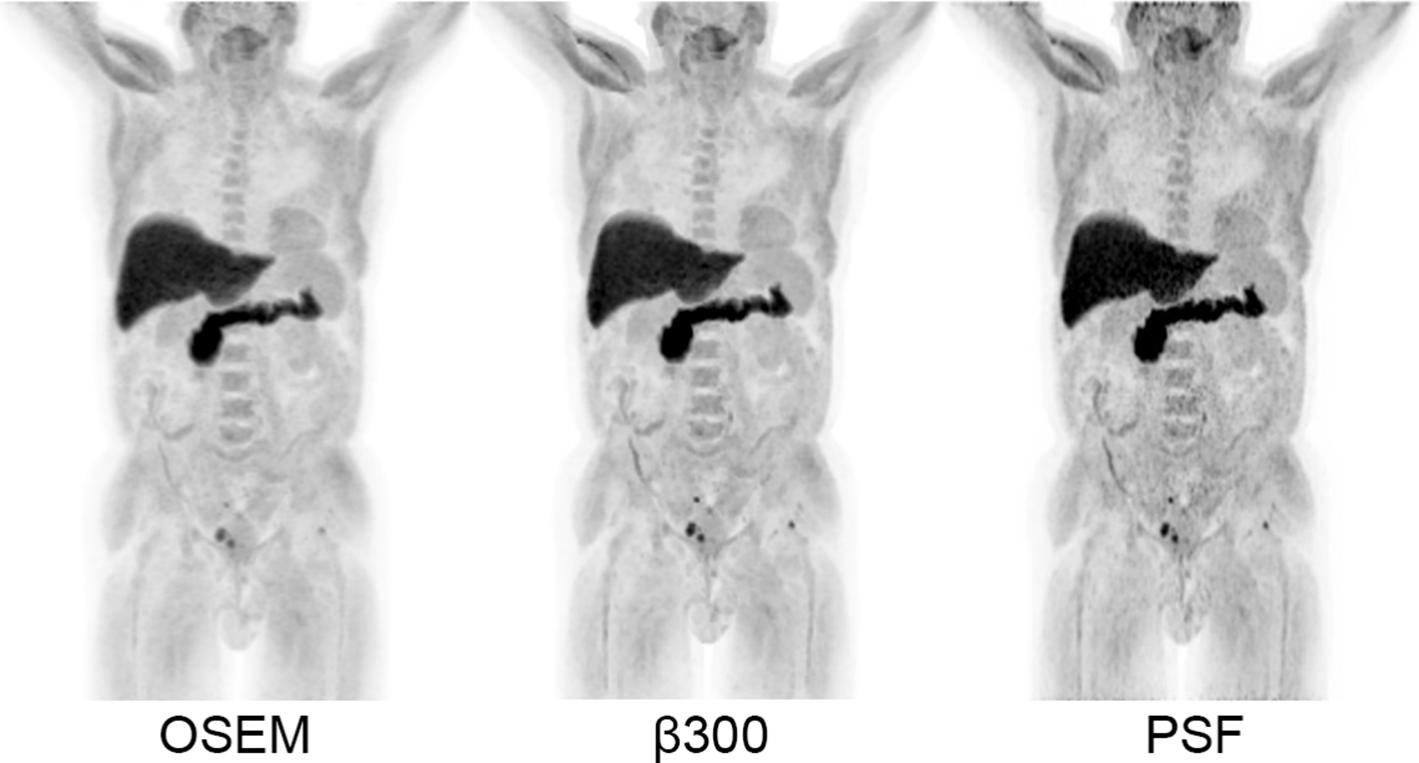
Maximum intensity projection images (SUV scale 0–7) of a patient demonstrating small foci of disease recurrence in the pelvis and left femoral head, rendered more conspicuous on the BPL β 300 reconstruction compared to OSEM, but without the penalty of increased noise depicted on the PSF reconstruction. OSEM, ordered subset expectation maximisation; PSF, point spread function; SUV, standardised uptake value.

β300 was indicated as the most preferred reconstruction in 9 out of 10 cases by one reader. On balance, the relatively minor difference in overall reader preference is deemed relatively immaterial to the strength of the other overarching findings. Therefore, β300 should be considered as the optimal penalisation factor for BPL reconstruction of ^18^F-fluciclovine PET images. A case example is presented in Figure 6.

Our findings are important because the use of newer PET reconstruction algorithms incorporating PSF modelling such as BPL may have implications for the interpretation of oncological PET/CT, particularly as it has been shown to increase sensitivity at a cost of reduced specificity for a given SUV threshold.^2^ Adoption of new reconstruction algorithms into well-established clinical practice, notably with interpreting ^18^F-FDG PET, demands dedication on the part of the reader to recalibrate their approach to reading studies. Widespread adoption of advanced reconstruction algorithms is challenging due to the highly variant experience with ^18^F-FDG PET reconstruction across the globe, which has been in constant iteration since the use of filtered back projection and 2D-mode scanning.

The landscape is encouragingly different with ^18^F-fluciclovine, which has recently been approved for a focused clinical indication (recurrent prostate cancer), with interpretation guidelines built on cumulative experience of a small consortia of readers using relatively modern scanner and reconstruction technology.^11, 12^ Furthermore, being an amino acid, it may be less fallible to non-specific uptake by inflammatory/benign entities compared to ^18^F-FDG as described *in vitro*,^13^ and anecdotally in published literature.^14, 15^

The difference in optimal β compared to ^18^F-FDG whole body PET, previously described to be β400,^1^ can be explained by the differences in physiological distribution and pathological uptake compared to ^18^F-fluciclovine. In prostate cancer, nodal recurrence tends to occur within the pelvis and retroperitoneum, where adjacent background uptake is relatively low. Improved demonstration of pathological uptake, particularly in small nodes, would then be afforded by a relatively low β, which generates increased signal at minimal penalty to surrounding background noise.

Being in the early stages of clinical rollout, there is an opportunity for naïve readers to gain their formative clinical experience with ^18^F-fluciclovine interpretation based on advanced image reconstruction from the start. Apart from affording a step-up in patient care, through improved image quality and confidence in interpretation, this should avoid the unnecessary complexities of relearning should current mainstream reconstruction technology get eclipsed.

Our experience with this process supports an ongoing collaborative effort amongst academic leaders and industry involved in this and other novel radiotracers, to maintain clear interpretation guidelines based on collective experience with different reconstruction technologies. This has to be supported by prospective study of diagnostic performance based on advanced reconstruction technology, and retrospective evaluation of the like where possible, using the same approach as this study, exploiting saved sinogram data. We also propose these actions for forthcoming imaging agents as they attain regulatory approval.

There are limitations to consider in this study. It was not possible to completely blind the scorers to the different reconstruction algorithms, as BPL has a different visual appearance compared to PSF at all -values, and to OSEM to a limited extent. This may have introduced bias as readers progressed through the scoring process, mitigated to some extent by the wide range of BPL reconstructions used. Finally, caution should be adopted in the event of expanded clinical use for ^18^F-fluciclovine, and revisiting optimisation of image reconstruction based on disease type and/or organ of interest, particularly the brain, may be warranted.

## Conclusion

BPL reconstruction of ^18^F-fluciclovine PET images improves SNR, affirmed by overall reader preferences for BPL reconstructions. On balance, β300 is suggested for ^18^F-fluciclovine whole body PET image reconstruction using BPL.
